# Supplementary material for: Waist Circumference and All-Cause Mortality among Older Adults in Rural Indonesia
Source: Int J Environ Res Public Health. 2019 Jan 3;16(1):116. doi: 10.3390/ijerph16010116 (PMC6339011; doi:10.3390/ijerph16010116)
Supplement: Supplementary file 1 [file ijerph-16-00116-s001.pdf]

IJERPH-398908
International Journal of Environmental Research and Public Health
Waist Circumference and All-Cause Mortality among Older Adults in Rural Indonesia
Cahya Utamie Pujilestari, Lennarth Nyström, Margareta Norberg, Nawi Ng

Supplementary Materials

Table S1. All-cause mortality stratified by sex and wealth status, with follow-up to 36 months.

	N	Number of Deaths	Percentiles							
			5th		25th		75th		95th	
			Waist (cm)	HR (95% CI)	Waist (cm)	HR (95% CI)	Waist (cm)	HR (95% CI)	Waist (cm)	HR (95% CI)
**Overall analysis on all samples with self-reported chronic diseases controlled as a covariate in the regression analysis (N = 10,997)<br>As shown in Table 2 in the manuscript**										
**Men**										
**Poor**	2,521	270	64	2.06 (1.28, 3.31) *	70	1.51 (0.94, 2.41)	80	1.27 (0.83, 1.94)	88	1.06 (0.91, 1.22)
**Rich**	2,608	241	65	1.01 (0.67, 1.54)	72	1.00 (0.69, 1.47)	83	0.92 (0.74, 1.16)	93	1.01 (0.92, 1.12)
**Women**										
**Poor**	3,141	269	62	1.36 (0.97, 1.90)	69	1.39 (1.06, 1.82) *	81	0.98 (0.93, 1.03)	92	0.89 (0.64, 1.25)
**Rich**	2,727	201	63	1.32 (0.86, 2.03)	72	1.22 (0.89, 1.66)	85	0.98 (0.77, 1.24)	97	1.24 (0.78, 1.98)
**Subgroup analysis after excluding individuals WITH self-reported chronic diseases (N = 8945)<br>Using the same cut-off points as in the overall analysis.**										
**Men**										
**Poor**	2,121	189	64	1.56 (0.88, 2.78)	70	1.26 (0.73, 2.17)	80	1.21 (0.67, 2.21)	88	1.14 (0.73, 1.77)
**Rich**	2,114	139	65	0.92 (0. 61, 1.40)	72	0.92 (0.61, 1.40)	83	0.99 (0.73, 1.35)	93	1.01 (0.84, 1.20)
**Women**										
**Poor**	2,595	177	62	1.33 (0.87, 2.03)	69	1.46 (0.96, 2.22)	81	0.96 (0.88, 1.04)	92	0.67 (0.43, 1.04)
**Rich**	2,115	129	63	0.94 (0.56, 1.57)	72	1.12 (0.84, 1.48)	85	0.95 (0.71, 1.28)	97	1.44 (0.82, 2.52)

Note: Hazard ratio (HR) and 95% confidence intervals (CI) from multivariate adjusted Cox regression analysis with restricted cubic splines of waist circumference at 5th, 25th, 75th and 95th percentiles. WHO-INDEPTH SAGE Purworejo longitudinal data (2007–2010).

Table S2. All-cause mortality stratified by sex and wealth status, with follow-up to 36 months and excluding all deaths happened during Month 1–23 of the follow-up.

	N	Number of Deaths	Percentiles							
			5th		25th		75th		95th	
			Waist (cm)	HR (95% CI)	Waist (cm)	HR (95% CI)	Waist (cm)	HR (95% CI)	Waist (cm)	HR (95% CI)
**Overall analysis on all samples with self-reported chronic diseases controlled as a covariate in the regression analysis (N = 10,316)**										
**Men**										
**Poor**	2,322	71	64	5.83 (1.41, 24.1) *	70	4.11 (1.06, 16.0) *	80	2.61 (0.55, 12)	88	1.45 (0.51, 4.14)
**Rich**	2,439	72	65	1.74 (0.89, 3.41)	72	1.27 (0.71, 2.29)	83	0.99 (0.77, 1.27)	93	1.53 (0.33, 7.15)
**Women**										
**Poor**	2,961	89	62	1.20 (0.66, 2.18)	69	1.35 (0.75, 2.44)	81	0.98 (0.79, 1.22)	92	0.43 (0.18, 1.01)
**Rich**	2,594	68	63	0.76 (0.34, 1.70)	72	1.11 (0.76, 1.63)	85	1.14 (0.94, 1.37)	97	3.14 (0.77, 12.8)
**Subgroup analysis after excluding individuals WITH self-reported chronic diseases (N = 8397)<br>Using the same cut-off points as in the overall analysis.**										
**Men**										
**Poor**	1,955	43	64	4.16 (0.62, 27.8)	70	4.11 (0.69, 24.4)	80	2.57 (0.27, 24.8)	88	1.21 (0.11, 12.8)
**Rich**	1,996	46	65	1.05 (0.48, 2.26)	72	0.71 (0.35, 1.42)	83	0.88 (0.57, 1.36)	93	1.21 (0.95, 1.56)
**Women**										
**Poor**	2,442	66	62	1.38 (0.71, 2.69)	69	1.25 (0. 62, 2.50)	81	1.02 (0.87, 1.21)	92	0.56 (0.24, 1.30)
**Rich**	2,004	49	63	0.56 (0.19, 1.63)	72	1.10 (0.70, 1.74)	85	0.78 (0.46, 1.34)	97	1.20 (0.47, 3.04)

Note: Hazard ratio (HR) and 95% confidence intervals (CI) from multivariate adjusted Cox regression analysis with restricted cubic splines of waist circumference at 5th, 25th, 75th and 95th percentiles. WHO-INDEPTH SAGE Purworejo longitudinal data (2007–2010).
